# Phonological and lexical influences on phonological awareness in children with specific language impairment and dyslexia

**DOI:** 10.3389/fpsyg.2014.00838

**Published:** 2014-08-04

**Authors:** Kelly Farquharson, Tracy M. Centanni, Chelsea E. Franzluebbers, Tiffany P. Hogan

**Affiliations:** ^1^Department of Special Education and Communication Disorders, University of Nebraska-LincolnLincoln, NE, USA; ^2^Crane Center for Early Childhood Research and Policy, Ohio State UniversityColumbus, OH, USA; ^3^Department of Communication Sciences and Disorders, MGH Institute of Health ProfessionsBoston, MA, USA

**Keywords:** dyslexia, specific language impairment, phonological awareness, neighborhood density, sound similarity

## Abstract

Children with dyslexia and/or specific language impairment have marked deficits in phonological processing, putting them at an increased risk for reading deficits. The current study sought to examine the influence of word-level phonological and lexical characteristics on phonological awareness. Children with dyslexia and/or specific language impairment were tested using a phoneme deletion task in which stimuli differed orthogonally by sound similarity and neighborhood density. Phonological and lexical factors influenced performance differently across groups. Children with dyslexia appeared to have a more immature and aberrant pattern of phonological and lexical influence (e.g., favoring sparse and similar features). Children with SLI performed less well than children who were typically developing, but followed a similar pattern of performance (e.g., favoring dense and dissimilar features). Collectively, our results point to both quantitative and qualitative differences in lexical organization and phonological representations in children with SLI and in children with dyslexia.

## Introduction

Phonological awareness skills have a causal influence on reading achievement (Foy and Mann, 2001; Catts et al., 2002; Hogan et al., 2005; Nancollis et al., 2005). Children with specific language impairment (SLI) and/or dyslexia are reported to have marked weaknesses in phonological awareness tasks (Catts et al., 2005). Subsequently, these children often experience significant difficulties with reading. Word-level phonological and lexical features influence performance on phonological tasks such as word learning (Storkel, 2003, 2009), nonword repetition (Vitevitch et al., 1997), and phonological awareness (Hogan, 2010) in both adults (Vitevich and Luce, 1998; Storkel et al., 2006) and children (Storkel and Rogers, 2000; Storkel, 2002; Stokes et al., 2012). Phonological features refer to the individual sounds and sound characteristics of words; lexical features refer to the holistic combination of sounds in words and their likeness to other words. Two prominent theories substantiate the influence of phonological and lexical features on phonological awareness: the phonological deficit hypothesis (Elbro, 1996) and the lexical restructuring model (Metsala and Walley, 1998). The phonological deficit hypothesis focuses on the influence of phonological features on phonological awareness. This theory posited that children with poor phonological awareness skills have difficulty storing and processing sounds in words. The lexical restructuring model focuses on the influence of lexical features on phonological awareness. This theory proposes that holistic mental representations of words become more and more detailed as a child's vocabulary grows. This phonemic detail helps to discern similar words from one another. As such, the larger a child's vocabulary is, the more phonemic detail is needed to distinguish similar words.

One phonological feature shown to influence phoneme awareness is sound similarity (Hogan, 2010), or the quantity of common phonemes within target words. For example, the phonemes /t/ and /d/ differ by only one distinctive feature (i.e., voicing), whereas the phonemes /m/ and /s/ differ by many (e.g., voicing, nasality, continuance, stridency, sonority, etc.). Hogan (2010) found, in an odd-one-out phoneme awareness task, that sounds that were similar to each other were more difficult to discriminate than sounds that were dissimilar. Another way to quantify sound similarity is by determining the sonority of co-occurring sounds. Sonority is a metric of how vowel-like a consonant phoneme is, based on the constriction of the vocal tract (Clements, 1992). Sonority is a particularly useful metric of sound similarity when the goal is to quantify phonological influences on a phoneme awareness task that involves deleting a sound instead of contrasting two sounds. Phoneme deletion tasks are most closely aligned to reading skills (Hogan et al., 2005). It has been posited that, due to coarticulation, sounds that are more sonorous, or similar, are difficult to delete from a word because they tie closely with the subsequent vowel (Yavas and Core, 2001). For instance, in a phoneme deletion task, a child may have more difficulty deleting the /l/ from the word “lap” than (s)he would deleting the /k/ from the word “cap” because /l/ is more sonorous, or similar to the vowel and /k/ is dissimilar to the vowel.

In addition to phonological features, lexical features have also been shown to affect phonological awareness. Specifically, neighborhood density is a lexical feature that quantifies the phonological similarity of words by calculating the number of related items to a target word allowing for the deletion, substitution, or omission of one phoneme. Target words that have many similar sounding “neighbors” are said to reside in a *dense* phonological neighborhood. Conversely, target words that have few similar sounding neighbors reside in a *sparse* phonological neighborhood. For example, a word such as “cat” has many similar sounding neighbors (e.g., cap, rat, cut, cats, mat, fat, pat, cot, etc.) whereas a word such a “these” has fewer neighbors (e.g., thee, ease, etc.).

In typically developing children, Storkel and colleagues have found that dense words are learned and maintained more readily than sparse words (Storkel and Adlof, 2009; Hoover et al., 2010). However, in children with poor phonological processing, neighborhood density appears to have a different influence (Storkel et al., 2010). This finding revealed that children with phonological delays continue to have difficulty creating lexical representations for new words, even with repeated exposure, and these children do not benefit from neighborhood density in the same way that typically developing children do. Considering that children with dyslexia also have poor phonological processing, we predicted that they may also show aberrant patterns of lexical influence on a phonological awareness task.

A recent study found that similar sounds were more difficult to discriminate in sparse words compared to dense words (Hogan et al., in press). Their results point to the importance of considering both phonological and lexical influences on phoneme awareness tasks. Additionally, these results substantiate the need to examine how word-level characteristics differentially influence children with phonological and lexical deficits. For the purposes of the current investigation, we examined phoneme deletion in children with SLI and children with dyslexia.

The phonological deficit hypothesis and the lexical restructuring model also lend support for the need to examine phonological and lexical influences in children with SLI and children with dyslexia. According to the phonological deficit hypothesis, children with dyslexia have problems perceiving and/or storing phonological information, which disrupts their formation of lexical representations. These poorly specified representations negatively impact phoneme awareness (Catts, 1986; Elbro et al., 1994; Elbro, 1996). This idea has been supported by numerous studies showing that children with dyslexia are less accurate at discriminating, identifying, and/or repeating the sounds in words (Brady et al., 1983; Kamhi et al., 1990). It is possible that phonological characteristics will influence phoneme awareness differently in children with dyslexia compared to their typical peers because their lexicons are structured differently. Moreover, children with dyslexia may not benefit from lexical restructuring in the same way as typically developing children. Metsala (1997) showed that children with dyslexia required more phonetic information to identify words from sparse neighborhoods compared to their typically developing peers. It is plausible that deficient phonological representations, predicted by the phonological deficit hypothesis, were underlying the apparent lexical differences. It is also a possibility that children with dyslexia may benefit from lexical restructuring in a similar manner as their typically developing peers, especially if they have intact language abilities.

Children with SLI have smaller lexicons and are at great risk to become reading impaired (Catts et al., 2005). Stokes (2010) and Stokes et al. (2012) found that children with smaller lexicons preferred dense words over sparse words. This suggests there could be a quantitative difference in how children with speech and language impairments learn and store new vocabulary words (but see Munson et al., 2005a). Based on the extant literature, we hypothesized that children with SLI, who do not have dyslexia, would show quantitatively, but not qualitatively, different patterns of phonological and lexical influences on phoneme awareness when compared to their typically developing peers and those with dyslexia.

Informed by well-known theories of phonological processing and reading development, the current study examined the influence of phonological and lexical features of words on a phoneme deletion task in children with SLI and dyslexia compared to their typical peers. We hypothesized that there would, indeed, be differences across the groups such that children with SLI would show a similar pattern of lexical and phonological influences on phoneme deletion to their typically developing peers; however due to their smaller lexicons, they would have weaker phoneme awareness than their peers. Children with dyslexia, we hypothesized, would show an aberrant pattern of phonological and lexical influences compared to their typically developing peers, which would signify underspecified, weak phonological and lexical representations.

## Methods

This research was conducted in conformity with the ethical standards of the field. Additionally, all aspects of this work were approved by the Institutional Review Board of the University of Nebraska-Lincoln.

### Participants

Participants included 64 children, aged 6;9–9;0, attending 2nd grade in both private and public school in the Midwest region of the United States. To reduce the effects of varying phonological and lexical representations within the groups, all children were monolingual, native English speakers. The children's primary spoken language was determined per parent report (Gutierrez–Clellen and Kreiter, 2003) and confirmed through interactions with each child. All children passed a hearing screening (ASHA, 1997) to rule out hearing impairment. To examine both typical and atypical phonological awareness development, the 64 participants were comprised of three groups: typically developing (TD; *N* = 33), children with specific language impairment (SLI; *N* = 13), and children with dyslexia (DYX; *N* = 18). Of note, the DYX group was comprised of children with dyslexia with varying language abilities. Only six of the 18 children with dyslexia had good language skills. As specified in our research questions, we were interested in examining the effect of a phonological deficit on phonological deletion performance in children with dyslexia. Thus, collapsing these two groups (e.g., pure dyslexia and dyslexia with comorbid SLI) allowed us to examine the differences between children with a language impairment in isolation and children who had a reading impairment. Unfortunately we did not have enough participants to create four groups with a double dissociation between language and word reading. Our difficulty recruiting those with dyslexia with good language was not surprising considering the high correlation between language and word reading skills in children with dyslexia (Catts, 1993; Bishop and Snowling, 2004; Catts et al., 2005). However, to shed light on the influence of language in our group with dyslexia, we ran *post-hoc* analyses comparing those with dyslexia only compared to their peers with co-morbid SLI and dyslexia.

### Procedures

Each child completed a battery of language, word decoding, nonverbal intelligence, and phonological awareness assessments during in the fall of the academic year. The measures included both standardized assessments and experimental tasks. Assessments were blocked into approximately 45 min time periods and administered in a predetermined order. Prior to testing, all assessors established reliability on administration of assessments and use of the equipment through one-on-one training sessions with a trained research assistant. Assessors completed training, observed sessions, and were observed in the field prior to testing independently. All assessment data was double scored in the research lab followed by double entry into the database to ensure accuracy of data.

#### Inclusionary measures

Measures assessing the following constructs were selected to address the study's main research questions: (1) phonological processing, (2) word decoding abilities, (3) language skills, and (4) nonverbal intelligence (Table 1). Inclusionary criterions used are well-documented tests of language, reading, and cognitive skills in children: two subtests of the Comprehensive Test of Phonological Processing (CTOPP; Wagner et al., 1999): Elision and Blending, two subtests of the Woodcock Reading Mastery Test, Revised (WRMT-R; Woodcock, 1987): Word Identification and Word Attack, The Clinical Evaluation of Language Fundamentals, Fourth Edition (CELF-4; Semel et al., 2003), and The Reynolds Intellectual Assessment Scales (RIAS; Reynolds and Kamphaus, 2002). To increase the validity of our sample, we set our cutoff points to eliminate any overlap between groups. Specifically, for inclusion in the DYX group, a WRMT-R standard score of 93 or below was required; for inclusion in the SLI group, a WRMT-R score of 100 or below and a CELF-4 score of 85 or below was required. All children had RIAS standard scores of 75 or higher in order to be included in the study.

**Table 1:** **Standardized scores and criteria for inclusion in each experimental group**.

	**WRMT^a^**	**EVT^b^**	**RIAS^c^**	**CTOPP^d^**	**CELF^e^**
TD (*N* = 33)	115.72 (1.57)	108.24 (1.70)	107.67 (2.61)	106.84 (1.93)	104.65 (1.52)
DYX and comorbid (*N* = 18)	86.50 (2.24)^***^	93.67 (1.79)^***^	101.56 (3.07)	93.50 (1.97)^***^	79.00 (2.71)^***^
SLI (*N* = 13)	106.23 (1.70)^***^	93.15 (3.25)^***^	99.84 (5.19)	93.76 (2.38)^***^	75.15 (2.05)^***^

a *Woodcock Reading Mastery Test (DYX/comorbid WRMT > 80, TD and SLI ≥ 100; Woodcock, 1987)*,

b *EVT (EVT-2, TD Standard Score > 100, other groups open; cite)*,

c *Reynolds Intellectual Assessment Scales (RIAS, All groups > 75; (Reynolds and Kamphaus, 2002)*,

d *CTOPP*,

e *Clinical Evaluation of Language Fundamentals (CELF SLI and DYX/comorbid Standard Score ≤ 85; TD Standard Score ≥ 90; Semel et al., 2003); unpaired t-tests vs. TD*,

*** *p < 0.001*.

#### Experimental measures

The Hogan Deletion Task (2010) was created to represent a range of phonological awareness difficulty in an attempt to minimize the possibility of floor and ceiling effects (Schatschneider et al., 1999) and provide a platform for contrasting sound similarity and neighborhood density. In this task, the child was asked to repeat a single-syllable word or nonword after he/she heard it and then say the word again with one sound deleted from the word. Examples are contained in Table 2. The phoneme deletion task was comprised of two types of real words and two types of nonwords: (1) those that required initial sound deletion and (2) those that required final sound deletion (note that for the purposes of the current investigation, initial and final sound conditions were collapsed). Within both subtests, words varied orthogonally by two dimensions: sound similarity and neighborhood density. Thus, a real word or nonword occurred in one of four conditions: similar-dense, similar-sparse, dissimilar-sparse, and dissimilar-dense, for a total of 80 items (Table 3).

**Table 2:** **Real word and non-word sample stimuli for the Hogan Deletion Task**.

**Condition**	**Repetition stimuli (deletion stimuli)**	**Sounds contrasted**	**Neighborhood density**
Similar-Dense (real words)	hat (at)	h/vowel	29
Similar-Dense (non-words)	yeet (eat)	j/vowel	15
Dissimilar-Sparse (real words)	table (able)	t/vowel	7
Dissimilar-Sparse (non-words)	kear (ear)	k/vowel	6
Similar-Sparse (real words)	wheel (eel)	w/vowel	6
Similar-Sparse (non-words)	won (on)	w/vowel	8
Dissimilar-Dense (real words)	chair (air)	ch/vowel	18
Dissimilar-Dense (non-words)	taan (on)	t/vowel	20

**Table 3:** **All possible combinations of stimuli during the task**.

**Which sound is deleted:**	**Initial sound deletion**	**Initial sound deletion**	**Final sound deletion**	**Final sound deletion**
Real or non-words	Real words	Non-words	Real words	Non-words
5 stimuli each:	Similar/dense	Similar/dense	Similar/dense	Similar/dense
5 stimuli each:	Dissimilar/dense	Dissimilar/dense	Dissimilar/dense	Dissimilar/dense
5 stimuli each:	Similar/sparse	Similar/sparse	Similar/sparse	Similar/sparse
5 stimuli each:	Dissimilar/sparse	Dissimilar/sparse	Dissimilar/sparse	Dissimilar/sparse
Totals:	20 stimuli	20 stimuli	20 stimuli	20 stimuli

*For each of two tasks (initial or final sound deletion), 20 stimuli were presented, which varied by sound similarity and neighborhood density. All stimuli were randomized and all children heard all 80 words*.

Six practice items were administered at the beginning of the Hogan Deletion Task. During the practice items, the examiner provided corrective feedback. To continue to the experimental task, the participant was required to complete 4 out of 6 of the practice items correctly. The practice set could be repeated up to 3 times, with the same 4 out of 6 criterion. If a child did not pass the practice items after 3 trials, he/she did not complete the experimental task. Our sample initially included 69 but, 5 children (comorbid, *n* = 2; typically developing, *n* = 3) did not pass the practice items and thus they did not continue on to complete the phoneme deletion task.

#### Stimuli

The stimuli for the Hogan Deletion Task consisted of individual real words and nonwords. Each real word and nonword duration was measured and ANOVAs were used to ensure that words in each condition did not differ. The real word stimuli were of high frequency (above 50) according to adult frequencies (Francis and Kucera, 1967) and child databases (Moe et al., 1982). Nonwords were created to match the real words on a word-by-word basis on word-level phonological and lexical characteristics. Real and nonword stimuli provided an internal control to account for possible influences of semantic information. To control for order effects, the items for each subtest were presented in random order. The presentation order of real words and nonwords was randomized between subjects. For the purposes of the current study, task performance was collapsed across real words and nonwords and across initial and final phoneme deletion conditions.

Sounds were contrasted according to the linguistic concept of sonority hierarchy/ranking (e.g., Clements, 1992). A sound's sonority rating refers to the level of constriction in the vocal tract during production (Chin, 1996). Yavas and Core (2001) reported that sounds that were more sonorous (e.g., /l/), were harder to delete at the end of a word compared to less sonorous sounds (e.g., /t/), which tended to be easier to delete (see also Treiman, 1989). The sonority effect is attributed to coarticulation; a more sonorous, or vowel-like, sound co-articulates more fluidly with its preceding vowel. In this study, more sonorous sounds were classified as similar, indicating similarity to the vowel. In contrast, less sonorous sounds were labeled dissimilar.

Stimuli were also manipulated by the lexical characteristic of neighborhood density. A phonological neighbor is a word that differs by one phoneme addition, deletion, or substitution. Neighborhood density was calculated by determining whether each of the test items had either many neighbors (i.e., >14; dense words) or few neighbors (i.e., <6; sparse words). Note that the values used to determine density of words is relatively arbitrary, but follows past research that has dichotomized neighborhood density in this way (Munson et al., 2005b; Hogan, 2010; Hoover et al., 2010). Neighborhood density was calculated using the Hoosier Mental Lexicon, a 20,000 word electronic database (Nusbaum et al., 1984). Nonword neighborhood density counts were based on the number of real word neighbors. Examples of real word and nonword stimuli from the phoneme deletion task can be found in Table 2 and Table 3 respectively. For these examples, the real words and nonwords are written using English orthography; however, regardless of the number of letters, the phonemic structure was consonant-vowel-consonant (CVC). Note that the participants did not see any orthography and only heard the stimuli via auditory recording.

#### Statistical analyses

Behavioral measures were analyzed for number of correct items per stimulus condition and repeated-measures ANOVAs were used to compare performance by stimulus condition across experimental groups as well as to test for interactions between stimulus similarity and neighborhood density. Unpaired t-tests were used to evaluate differences across groups in inclusion measures and test items (*p* < 0.05). Bonferroni correction was used to correct for any multiple comparisons.

### Results

#### Behavioral profile of each experimental group

In the current study, we evaluated 64 children who were generally matched on age and gender (average age: 7;9, unpaired *t*-tests across groups for age, *p* > 0.04; and gender ratio, *p* > 0.61) on a variety of speech, language, and intelligence measures. Standard scores on these measures (based on a population mean of 100 ± 15) were used to categorize the participants into three experimental groups (Table 1): typically developing (TD; *N* = 33), specific language impaired (SLI; *N* = 13) and a third group composed of those with dyslexia with and without SLI. (DYX; *N* = 18). Intelligence scores, measured by the Reynolds Intellectual Assessment Scales (RIAS; Reynolds and Kamphaus, 2002), did not differ among the three groups, whereas scores for all other measures were significantly lower for the DYX and SLI only groups when compared to TD controls (unpaired *t*-tests, *p* < 0.001; Table 1). These inclusionary criteria adhere to and are slightly stricter than previous studies of these groups. Previous studies using these participant groups have employed much broader inclusion criterion than we used here and often overlap the cutoff points or do not report cutoff points at all (Ramus et al., 2003; Rispens and Been, 2007). The increased control over inclusion criterion in our study increases the validity of this sample for comparison between disordered groups.

#### Confirmation of previous task performance in TD children

All children completed a phonological awareness task that involved repetition of real and nonwords and deletion of an initial or final phoneme. Analyses are collapsed across word condition and phoneme position condition. Stimuli were orthogonally manipulated by sound similarity and neighborhood density (see Methods). Data were submitted to a repeated-measures ANOVA, with two within subjects factors (similarity: similar vs. dissimilar and density: dense vs. sparse). On the repetition portion of the task, there was a significant interaction between similarity and density [*F*_(1, 61)_ = 10.481; *p* = 0.002, η^2^_*p*_ = 0.147] as well as a between subjects main effect of group [*F*_(2, 61)_ = 7.52, *p* = 0.001, η^2^_*p*_ = 0.198]. On the deletion portion of the task, there was a significant interaction between similarity and density [*F*_(1, 61)_ = 21.855, *p* < 0.001, η^2^_*p*_ = 0.264] as well as a between subjects main effect of similarity [*F*_(1, 61)_ = 21.889, *p* < 0.001, η^2^_*p*_ = 0.264] and group [*F*_(2, 61)_ = 5.253, *p* < 0.01, η^2^_*p*_ = 0.147]. Several repeated measures ANOVAs were also completed for each group.

**Figure 1:**
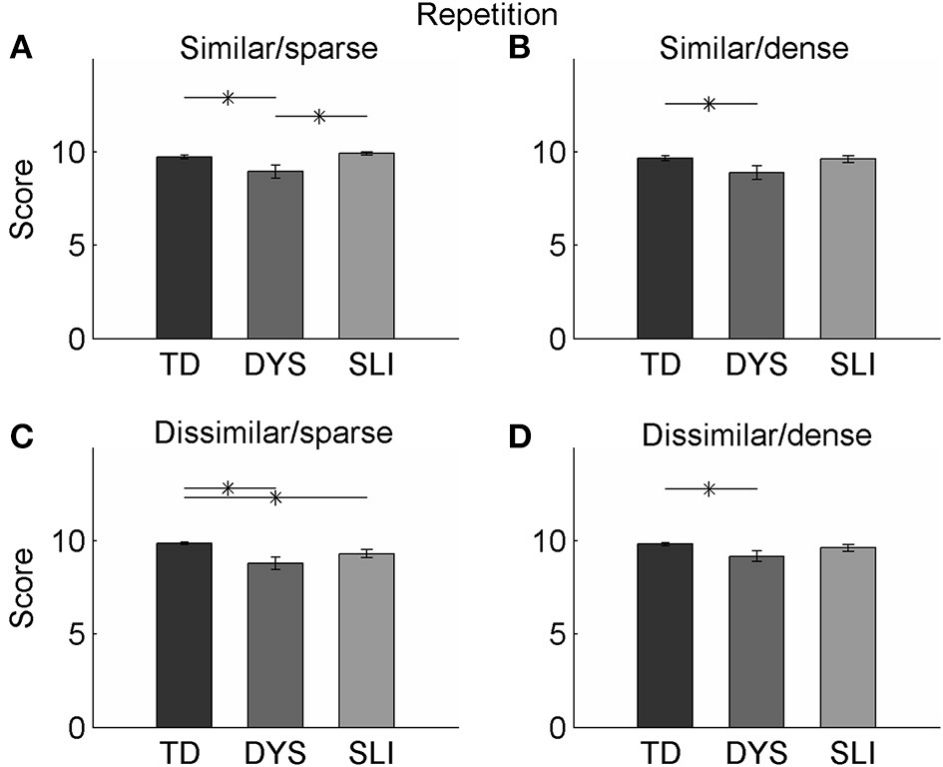
**Performance on the repetition portion of the task**. **(A)** DYS children performed worse than both TD and SLI children on the repetition portion of the task when words were from sparse neighborhoods and when the words were similar sounding. **(B)** All groups performed similarly on the task when words were from dense neighborhoods and were similar sounding, though the DYS group's performance was significantly below the TD group. **(C)** TD children performed better than both DYS and SLI children when words were from sparse neighborhoods and were dissimilar sounding. **(D)** All groups performed similarly on the task when words were from dense neighborhoods and were dissimilar sounding, though the DYS group's performance was significantly below the TD group.

As predicted, the typically developing children repeated words more accurately when the words were from dense neighborhoods and the first or last two sounds were similar (*M* = 9.85, *SD* = 0.36, s.e.m. = 0.01; out of 10 correct). They showed the lowest level of accuracy when repeating words from sparse neighborhoods when the first or last two sounds were dissimilar, though the difference in performance was not statistically significant (*M* = 9.67, *SD* = 0.74, s.e.m. = 0.08; out of 10 correct; paired *t*-test, *p* = 0.16). We did not find any significant main effects or interactions on the repetition portion of the task (*p* > 0.10). On the deletion portion of the task, TD children performed with the highest accuracy on sounds that were from dense neighborhoods and the first or last two sounds were dissimilar (*M* = 9.03, *SD* = 1.56, s.e.m. = 0.27; out of 10 correct). They showed the lowest level of accuracy when repeating words that were from dense neighborhoods and the first or last two sounds were similar (*M* = 8.30, *SD* = 1.59, s.e.m. = 2.8; out of 10 correct; paired *t*-test, *p* < 0.01). These results are in line with previous work on sound influences on phonological processing tasks in typically developing children (Metsala, 1997; Storkel et al., 2006). We found a significant main effect of similarity during the deletion tasks, such that children scored higher on dissimilar words compared to similar words, regardless of density [*F*_(1, 32)_ = 11.559, *p* = 0.002, η^2^_*p*_ = 0.265; Figure 1]. The result that performances of TD children on the Hogan Deletion Task are comparable to previous work provides validation for the use of our task to contrast typical development with performance by groups of children with disordered language and reading.

#### Performance on repetition and deletion by SLI and DYX groups

Children with SLI achieved the highest accuracy on the repetition portion of the task when words were from dense neighborhoods and when the first or last two sounds were dissimilar (*M* = 9.92, *SD* = 0.28, s.e.m. = 0.08; out of 10 correct). They showed the lowest level of accuracy when repeating words that were from dense neighborhoods and the first or last two sounds were similar (*M* = 9.31, *SD* = 0.75, s.e.m. = 0.22; out of 10 correct; paired *t*-test, *p* < 0.01; Figure 1). On this portion of the task, we did observe a significant interaction between similarity and density [*F*_(1, 12)_ = 8.348, *p* = 0.014, η^2^_*p*_ = 0.410], though performance levels on words with a sparse neighborhood density were the same regardless of similarity, which indicates an advantage on the repetition portion of the task for dissimilar words in this group. Compared to the TD group, the SLI group was only significantly different in one condition; the SLI group was significantly better than TD controls on similar/dense words (unpaired *t*-test, *p* < 0.01).

**Figure 2:**
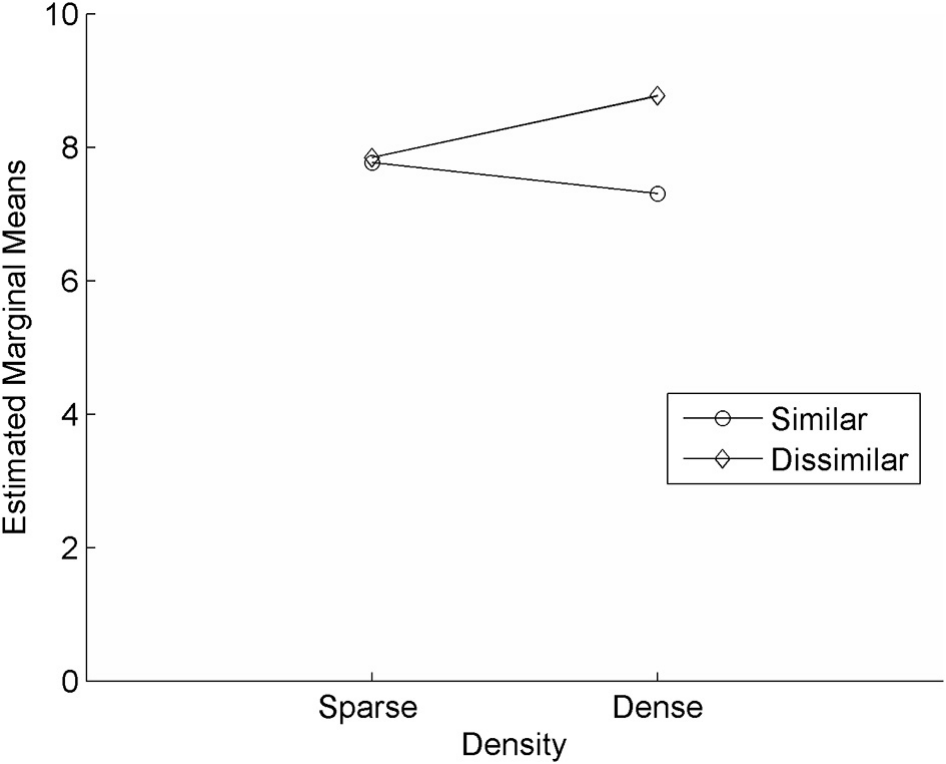
**Effect of similarity and density on deletion tasks in children with SLI**. Similarity did not seem to affect level of accuracy when words were from sparse neighborhoods. When words were from dense neighborhoods, similarity did play a significant role; dissimilar sounding words were easier than similar sounding words.

Just as with the repetition portion of the task, children with SLI achieved the highest accuracy when deleting phonemes from words that were from dense neighborhoods and when the first or last two sounds were dissimilar (*M* = 8.77, *SD* = 1.74, s.e.m. = 0.50; out of 10 correct). They showed the lowest level of accuracy when deleting phonemes from words that were from dense neighborhoods and when the first or last two sounds were similar (*M* = 7.31, *SD* = 1.38, s.e.m. = 0.39; out of 10 correct; paired *t*-test, *p* = 0.03). We again saw a significant interaction between similarity and density [*F*_(1, 12)_ = 5.635, *p* = 0.035, η^2^_*p*_ = 0.320] and on both deletion and repetition, density was more influential when the words were dissimilar (dissimilar/dense words were easier than dissimilar/sparse words; Figure 2). In general, performance trends of SLI kids were similar to TD children and the biggest contrast between these groups was the raw scores. These results suggest that there is a quantitative difference across groups, rather than a qualitative difference in phonological processing.

The group of children with DYX showed different trends on performance of the repetition portion of the task compared to the SLI only and TD groups (Table 4). Children in this group performed significantly worse than TD children on all four condition combinations (unpaired *t*-tests; similar/sparse, *p* < 0.01; similar/dense, *p* < 0.001; dissimilar/sparse, *p* = 0.01; dissimilar/dense, *p* < 0.01; Figure 1). The highest accuracy was achieved on the repetition portion of the task when the words were in sparse neighborhoods and when the first or last two sounds were similar (*M* = 9.85, *SD* = 0.65, s.e.m. = 0.34; out of 10 correct). They showed the lowest level of accuracy when repeating words that were from dense neighborhoods and when the first or last two sounds were similar (*M* = 8.89, *SD* = 0.75, s.e.m. = 0.37; out of 10 correct). Accuracy on this task was relatively consistent across the stimulus type (scores were within 0.4). There was a trend toward an interaction between similarity and density [*F*_(1, 17)_ = 3.676, *p* = 0.07, η^2^_*p*_ = 0.178) but did not reach significance, likely due to the even performance across stimulus conditions. This trend seemed to be driven by the influence of density on similar words. When words were similar, sparse neighborhood density increased accuracy.

**Table 4:** **Qualitative differences in highest and lowest accuracy scores by group and task**.

**TD**	**Repetition best**	**Repetition worst**	**Deletion best**	**Deletion worst**
	**Similar/dense**	**Dissimilar/sparse**	**Dissimilar/dense**	**Similar/dense**
*M*	9.85	9.67	9.03	8.3
*SD*	0.36	0.74	1.56	1.59
s.e.m.	0.01	0.08	0.27	2.8
**DYX/comorbid**	**Similar/sparse**	**Similar/dense**	**Dissimilar/dense**	**Similar/dense**
*M*	9.85	8.89	7.89	6.28
*SD*	0.65	0.75	2.35	2.08
s.e.m.	0.34	0.37	0.57	0.5
**SLI**	**Dissimilar/dense**	**Similar/dense**	**Dissimilar/dense**	**Similar/dense**
*M*	9.92	9.31	8.77	7.31
*SD*	0.28	0.75	1.74	1.38
s.e.m.	0.08	0.22	0.5	0.39

*There was no difference in the effect of similarity or density on scores on the Deletion tasks, but there were differences in the effects of similarity and density on the repetition portion of the task in the DYX/SLI and SLI only groups*.

**Figure 3:**
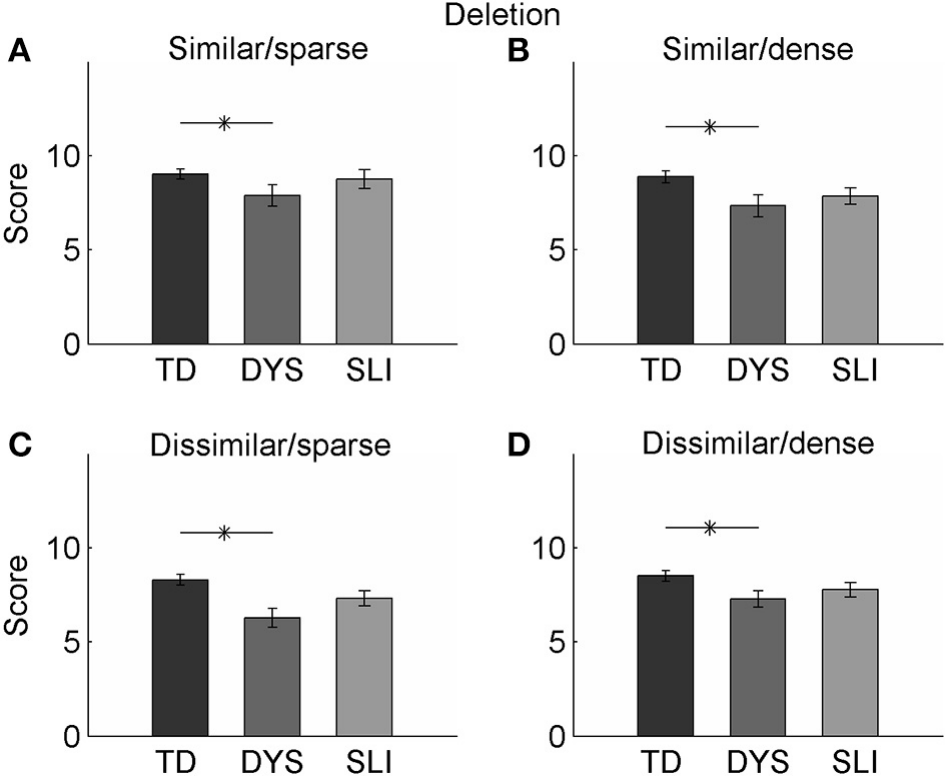
**Performance on the deletion portion of the task**. **(A–D)** On all combinations of stimuli on the deletion task, the DYX group scored significantly lower than the TD group. There were no significant differences between the SLI group and the TD group.

Children in the DYX group showed the same tendencies as children in the SLI and TD groups during deletion tasks: accuracy was highest on words from dense neighborhoods when the first or last two sounds were dissimilar (*M* = 7.89, *SD* = 2.35, s.e.m. = 0.57; out of 10 correct). They showed the lowest level of accuracy on words that were also from dense neighborhoods and when the first or last two sounds were similar (Table 4). Children in this group performed significantly worse than TD children on all four condition combinations (*M* = 6.28, *SD* = 2.08, s.e.m. = 0.50; out of 10 correct; unpaired *t*-tests, *p* < 0.04; Figure 3). There was a main effect of similarity [*F*_(1, 17)_ = 7.870, *p* = 0.01, η^2^_*p*_ = 0.316] and a significant interaction between similarity and density in this group during deletion tasks [*F*_(1, 17)_ = 11.858, *p* = 0.003, η^2^_*p*_ = 0.411]. These results suggest that children with DYX have qualitatively different patterns of performance on repetition and deletion tasks compared to children with SLI and TD children.

#### Effect of co-morbidity on task performance

**Figure 4:**
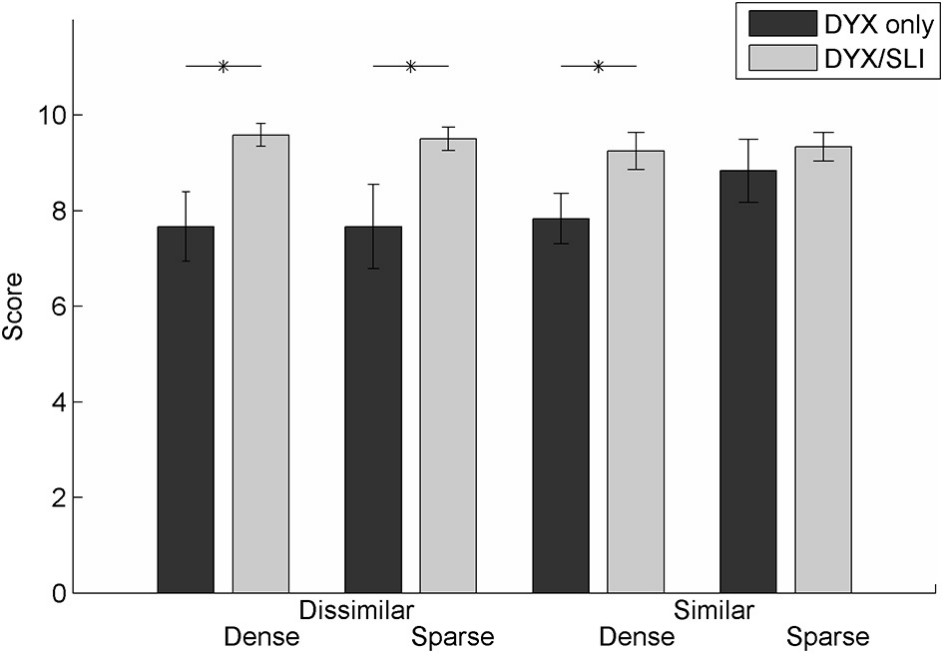
**Comparison of performance on the repetition portion of the task between DYX only and DYX/SLI children**. Children with dyslexia alone (DYX) displayed a different pattern of accuracy compared to children with dyslexia that were also comorbid for SLI (DYX). Children with DYX only has significantly lower accuracy on three of the four stimulus types (unpaired *t*-tests, dissimilar/dense, *p* < 0.01; dissimilar/sparse, *p* = 0.01; similar/dense, *p* = 0.03; similar/sparse, *p* = 0.40).

The DYX group in this study contained children that had dyslexia in isolation as well comorbidity with SLI. Due to the small number of children with DYX only (*N* = 6), we combined these children with those with dyslexia and SLI for the analyses described above. To determine whether co-morbidity played a role in the performance of this group, we evaluated task accuracy of those with dyslexia only (*N* = 6) and those with dyslexia and comorbid SLI (*N* = 12). Though we saw no differences across sub-groups on deletion tasks (unpaired *t*-tests, all comparisons *p* > 0.50), we did see significant differences between the dyslexia only and the participants with comorbid SLI and dyslexia on the repetition portion of the task. Three of the four condition combinations were significantly more difficult for the children with dyslexia only compared to the comorbid kids (dissimilar/dense, *p* < 0.01; dissimilar/sparse, *p* = 0.01; similar/dense, *p* = 0.03; similar/sparse, *p* = 0.40; Figure 4). These results suggest that the repetition task was sensitive to the influence of language variability in those with dyslexia. Note that the pattern of findings was the same as shown in prior analyses for children with dyslexia regardless of language impairment, however, interestingly, the children with dyslexia with good language skills had lower performance than their peers with co-morbid dyslexia and SLI. One plausible explanation for this finding is that the group of children with dyslexia-only had lower word reading abilities (WRMT standard score *M* = 83) compared to the children in the comorbid group (WRMT standard score *M* = 88. However, likely due to low power, the groups were not statistically significantly different in word reading (*p* = 0.32). Future studies should further explore the differences in phonological processing in a larger group of children with dyslexia with and without co-morbid language impairment.

## Discussion

The current study sought to explore the influence of sound similarity and neighborhood density on phonological awareness skills in children with SLI, dyslexia, and their typically developing peers. We hypothesized that there would, indeed, be differences across the groups such that children with SLI would show a similar pattern of lexical and phonological influences on phoneme deletion to their typically developing peers; however, due to their smaller lexicons, they would have weaker phoneme awareness performance than their peers. Children with dyslexia, we hypothesized, would show an aberrant pattern of phonological and lexical influences compared to their typically developing peers, which would signify underspecified, weak phonological and lexical representations.

Results yielded three major findings: (a) typically developing children experienced an advantage for dense and dissimilar words, (b) children with SLI showed a similar pattern of performance to children who were typically developing, across the phonological and lexical conditions, and (c) children with dyslexia exhibited an aberrant, immature pattern of performance when compared to children with SLI and typically developing peers.

First, in support of our hypothesis, children who were typically developing exhibited better phoneme repetition and deletion for words that were dense and dissimilar. This result is in line with previous research highlighting the density benefits on phonological tasks for typically developing children (Munson et al., 2005b; Hogan, 2010; Hoover et al., 2010). Specifically, the extant research indicates that children who are typically developing will recall dense words more readily than sparse and that they will more easily discriminate sounds that are dissimilar compared to similar. The current results support and expand these findings by examining performance on a phonological awareness task that required both word repetition and phoneme deletion. Similar to Hogan (2010), an interaction showed that typically developing children performed better on dissimilar sounds, regardless of the density of the word. Collectively, these results support both the lexical restructuring model and the phonological deficit hypothesis. That is, it appears based on the performance of children who are typically developing, that they have well-organized lexicons and more fine-grained and specified phonological representations.

Second, across the phonological and lexical conditions, children with SLI exhibited a similar pattern to their typically developing peers on both repetition and deletion. Although there has been little work examining the ways in which phonological and lexical factors influence performance in children with SLI, this result does support previous research (Stokes, 2010; Stokes et al., 2012). Specifically, Stokes (2010); Stokes et al. (2012) reported a dense advantage for children with small lexicons. Although their population was not identified has specifically having a language disorder, these children did exhibit smaller lexicons, which is a primary characteristic of children with SLI. These researchers, along with several others, suggested that dense words are easier to hold within memory, making it is easier to create a lasting lexical representation. Based on these results, it appears that children with SLI are quantitatively different from typically developing children, but not qualitatively different. We offer two possible explanations for this group difference. First, the ability to acquire and use new vocabulary words is circumscribed in children with SLI by difficulty storing new lexical representations. In accordance with the lexical restructuring model, the more words a child has stored in his/her lexicon, the more specified those words will become. Thus, children with SLI show a density benefit, but it is less robust than their typical peers because their lexicons are not as large. Second, a complimentary line of research has suggested that children with SLI struggle with word recall due to deficits in working memory (Gathercole and Baddeley, 1990; Ellis Weismer et al., 1999; Mainela-Arnold and Evans, 2005; Archibald et al., 2011). For example, Ellis Weismer and colleagues found that children with SLI performed poorer than typically developing peers on the word recall portion of a phonological working memory task. Thus, it is possible that children with SLI rely on neighborhood density to bootstrap into their limited lexicons, with dense words being easier to store. There is, however, evidence to the contrary (Mainela-Arnold and Evans, 2005), suggesting that future work should examine the nature of the role of working memory in lexical storage for children with SLI. Finally, keep in mind that the children with SLI in this sample scored in the normal range on word reading. Therefore, in terms of phonological processing, they are more like their typical peers than their peers with dyslexia.

Third, and perhaps most importantly, children with dyslexia exhibited the opposite pattern of phonological and lexical influences on phonological processing compared to their peers with SLI and their typically developing peers. Specifically, our sample of children with dyslexia showed the best performance on sparse words with similar sounds during repetition; on the deletion portion, they exhibited their best performance on dense words with dissimilar sounds. Extent literature consistently shows that children with dyslexia have weak phonological representations during word learning and picture naming tasks (Swan and Goswami, 1997; Elbro and Jensen, 2005). For instance, Elbro and Jensen (2005) found that 4–6th grade children with dyslexia scored poorer than 2nd grade children who were typically developing on a measure of quality of phonological representations. Specifically, children with dyslexia had difficultly accurately repeating and determining distinctions between multi-syllabic words. This suggests that dyslexia disrupts the ability to store, encode, and process phonological information in long-term memory. This disruption could be cognitively taxing and utilize all of the phonological resources that the child has, making it difficult to build a strong lexical representation. Previous research has reported that children with phonological delays favor sparse words over dense words in word learning paradigms (Storkel et al., 2010). As dyslexia is a phonological delay, albeit not one that always manifests as a speech production impairment, it was not surprising that the children with dyslexia benefitted differently from the phonological and lexical features of words. Thus, there is not only a quantitative difference between children who were typically developing, children with SLI, and children with dyslexia, but there is also a qualitative difference. In examining the patterns across the three groups, it appears that children with dyslexia exhibited an aberrant, immature pattern that is characteristic of less specified phonological representations. Storkel (2004) also suggests that underspecified phonological representations are the reason why children with phonological deficits do not benefit from phonological and lexical characteristics in the ways that children who are typically developing do. Underspecified, or weak, phonological representations have been strongly tied to poor word reading (Elbro, 1996; Swan and Goswami, 1997; Boada and Pennington, 2006) and lead to difficulty manipulating and producing the sounds within the ambient language.

## Limitations and conclusions

The present investigation is not without limitations. One primary limitation is that the speech production skills of the participants were not assessed. Specifically, if a child had difficulty with the production of a phoneme due to a speech sound disorder (e.g., /w/ substituted for /r/), then their performance on the repetition and deletion tasks may not necessarily reflect their phonological processing skills. Although most studies of phoneme awareness do not take into account the impact of speech errors, (Gillon, 2000; Munson et al., 2005a,b), future work should assess speech production skills to determine the effects of an expressive phonological disorder on scoring of phonological awareness tasks, especially considering the documented co-morbidity between speech and language impairment (Shriberg et al., 1999; Pennington and Bishop, 2009).

In conclusion, the results of the current investigation support the phonological deficit hypothesis, which suggests that children with dyslexia have underspecified phonological representations. Children with dyslexia, in this sample, exhibited difficulty with phoneme repetition and phoneme deletion compared to peers with SLI and those who were typically developing. Additionally, children with dyslexia appeared to have a more immature and aberrant pattern of phonological and lexical influence. Current results also support the lexical restructuring model, which suggests that children with SLI have limited lexicons that cause difficulty in storing words as fine-grained vs. holistic units. Children with SLI performed less well than children who were typically developing, but followed a similar pattern of performance. Collectively, our results point to both quantitative and qualitative differences in lexical organization and phonological representations in these groups of children. These findings may lead to the creation of new, data-driven, theory-based phonological awareness assessments using words most likely to detect change in children at risk for reading impairment.

### Conflict of interest statement

The authors declare that the research was conducted in the absence of any commercial or financial relationships that could be construed as a potential conflict of interest.
